# Rapid preparation of nuclei-depleted detergent-resistant membrane fractions suitable for proteomics analysis

**DOI:** 10.1186/1471-2121-9-30

**Published:** 2008-06-05

**Authors:** Rosalyn M Adam, Wei Yang, Dolores Di Vizio, Nishit K Mukhopadhyay, Hanno Steen

**Affiliations:** 1 Urological Diseases Research Center, Children's Hospital Boston and Department of Surgery, Harvard Medical School, Boston, MA 02115, USA; 2 Department of Pathology, Children's Hospital Boston and Harvard Medical School; Boston, MA, 02115, USA

## Abstract

**Background:**

Cholesterol-rich membrane microdomains known as lipid rafts have been implicated in diverse physiologic processes including lipid transport and signal transduction. Lipid rafts were originally defined as detergent-resistant membranes (DRMs) due to their relative insolubility in cold non-ionic detergents. Recent findings suggest that, although DRMs are not equivalent to lipid rafts, the presence of a given protein within DRMs strongly suggests its potential for raft association in vivo. Therefore, isolation of DRMs represents a useful starting point for biochemical analysis of lipid rafts. The physicochemical properties of DRMs present unique challenges to analysis of their protein composition. Existing methods of isolating DRM-enriched fractions involve flotation of cell extracts in a sucrose density gradient, which, although successful, can be labor intensive, time consuming and results in dilute sucrose-containing fractions with limited utility for direct proteomic analysis. In addition, several studies describing the proteomic characterization of DRMs using this and other approaches have reported the presence of nuclear proteins in such fractions. It is unclear whether these results reflect trafficking of nuclear proteins to DRMs or whether they arise from nuclear contamination during isolation. To address these issues, we have modified a published differential detergent extraction method to enable rapid DRM isolation that minimizes nuclear contamination and yields fractions compatible with mass spectrometry.

**Results:**

DRM-enriched fractions isolated using the conventional or modified extraction methods displayed comparable profiles of known DRM-associated proteins, including flotillins, GPI-anchored proteins and heterotrimeric G-protein subunits. Thus, the modified procedure yielded fractions consistent with those isolated by existing methods. However, we observed a marked reduction in the percentage of nuclear proteins identified in DRM fractions isolated with the modified method (15%) compared to DRMs isolated by conventional means (36%). Furthermore, of the 21 nuclear proteins identified exclusively in modified DRM fractions, 16 have been reported to exist in other subcellular sites, with evidence to suggest shuttling of these species between the nucleus and other organelles.

**Conclusion:**

We describe a modified DRM isolation procedure that generates DRMs that are largely free of nuclear contamination and that is compatible with downstream proteomic analyses with minimal additional processing. Our findings also imply that identification of nuclear proteins in DRMs is likely to reflect legitimate movement of proteins between compartments, and is not a result of contamination during extraction.

## Background

Lipid rafts and caveolae, membrane microdomains that are enriched in cholesterol and sphingolipids, have been implicated in diverse physiologic mechanisms, such as signal transduction, trafficking and lipid transport [1,2]. The invaginated and vesicular structures known as caveolae are a type of microdomain that harbor caveolin family proteins; however, non-caveolar 'flat' lipid rafts have also been shown to exist in cells that do not express caveolins [3]. Lipid rafts were originally defined as detergent-resistant membranes (DRMs) because of their relative insolubility in cold non-ionic detergents [4]. However, equating DRMs with lipid rafts is now believed to be inaccurate due to controversy over the biophysical and biochemical nature of rafts, and whether they even exist in vivo [3,5-7]. In addressing these concerns, several recent papers have proposed a revision of the raft hypothesis. These studies suggest that, although DRMs do not correspond to lipid rafts as they exist in vivo, the presence of a given protein within a DRM, and its loss from that fraction when DRMs are perturbed by cholesterol depletion, for example, strongly suggests the potential for raft association of that protein in vivo [8-10]. In addition, it has been noted in a recent publication that, despite its limitations, isolation of DRMs is the only biochemical approach for assessing protein interactions with rafts [10].

DRMs are thought to be diverse in protein content and in functional roles. However, these membrane domains are still poorly understood and are currently being characterized using proteomics tools [11]. The cell signaling function of DRMs is thought to arise from the ability of these microdomains to selectively retain or exclude specific proteins, resulting in the formation of multiprotein complexes that process biochemical information across specific signaling axes. Because cholesterol-rich microdomains may be functionally altered by pathophysiologic alterations in lipid metabolism [12], systematic analysis of proteins present in DRMs is likely to provide novel insights into multiple signaling mechanisms that operate in the normal state and in disease.

The biophysical and biochemical properties of DRMs present unique challenges to studies of their protein composition. Membrane proteins possess hydrophobic regions that render them poorly soluble during extraction [13,14]. In addition, DRMs represent a minor and transient component of the total membrane surface. The classical method of isolating DRM-enriched fractions is by flotation of cell extracts, prepared in cold detergents such as Triton X-100 or under detergent-free conditions, in a sucrose density gradient [15]. This approach exploits the detergent insolubility of DRMs at low temperatures as well as their light buoyant density. However, gradient centrifugation procedures are time-consuming and labor-intensive, requiring processing times on the order of 24 h. Moreover, the resultant dilute, sucrose-containing fractions have limited utility for proteomic analysis without additional processing steps [16].

We have employed a DRM isolation technique originally described by Solomon *et al*. [17,18] that exploits the differential solubility of detergent-resistant microdomains in cold, non-ionic detergents [19-24]. This approach is rapid and yields fractions that are comparable to those isolated by flotation on density gradients. We have validated our observations from the detergent extraction procedure using alternative approaches, including sucrose density ultracentrifugation and immunofluorescence imaging [21,23,24]. Both unpublished and published data from us and others revealed the presence of nuclear proteins in DRM fractions isolated either by flotation in density gradients or by differential detergent solubility [16,25-30](N.K.M. and R.M.A, unpublished observations). However, whether this unanticipated finding arose from intracellular trafficking of proteins between nuclei and DRMs, or, alternatively, was a reflection of nuclear contamination during extraction has not been determined. In this study, we describe a modification of the existing differential detergent extraction procedure that minimizes nuclear contamination of DRMs. Proteomic analysis of the resulting fractions suggests that the presence of 'nuclear' proteins in DRMs is likely to result from shuttling between the nucleus and other subcellular sites.

## Results and discussion

**Figure 1:**
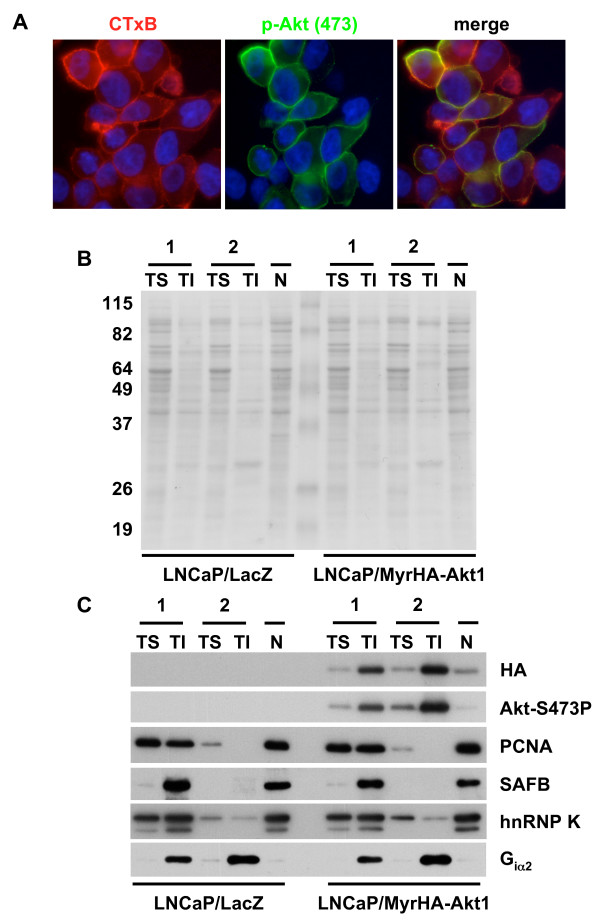
**Modification of the detergent extraction procedure eliminates 'nuclear' proteins from detergent-resistant membranes**. **(A) **Cholesterol- and sphingolipid-enriched membranes in LNCaP/MyrAkt1 cells were stained with 0.5 μg/mL Alexa 594-CTxB for 10 min prior to staining with anti-S473-P Akt (1:100) and FITC-conjugated secondary Ab (1:100). Nuclei were counterstained with DAPI prior to imaging. Original magnification, 63×. LNCaP cells stably expressing LacZ or MyrAkt1 were extracted using the conventional (1) or modified (2) detergent extraction procedure. Equal amounts (30 μg for detection of SAFB; 10 μg for other target proteins) of Triton-soluble (TS), Triton-insoluble, octylglucoside-soluble (TI) or nuclear (N) fractions were resolved by SDS-PAGE, transferred to nitrocellulose membranes and **(B) **stained with Ponceau S or **(C) **blotted with antibodies to the HA epitope tag, phospho-S473 Akt, PCNA, SAFB, hnRNP K and G_iα2_.

To investigate whether the presence of nuclear proteins in DRMs was an artifact or instead reflected physiologic trafficking events, we modified the extraction procedure described by Solomon *et al. *by pelleting out nuclei and intact cells from detergent-free homogenates prior to detergent solubilization. We reasoned that this would reduce potential contamination from nuclear material. As a model system, we have used caveolin-negative LNCaP prostate cancer cells stably expressing myristoylated Akt1 (MyrAkt1)[24]. MyrAkt1 is a robust marker of DRMs in these cells based on (i) its presence in light buoyant density fractions following sucrose gradient centrifugation; (ii) its enrichment in DRMs following differential detergent extraction; and (iii) its accumulation at the plasma membrane based on localization to membranes that stain with CTxB, a marker of the DRM/lipid raft-restricted ganglioside GM1 (Figure 1A)[31].

To determine the effect of pelleting nuclei and/or intact cells on the overall protein profiles of isolated fractions, we initially compared the conventional extraction method with the modified procedure. As starting material for the extraction, we generated membrane preparations from LNCaP/MyrHA-Akt1 or LNCaP/LacZ cells in log-phase growth by mechanical disruption of cells under detergent-free conditions, as described in Methods. In the conventional method, homogenized samples were centrifuged at 16,000 × *g *in a Beckman microcentrifuge for 10 min at 4°C. In the modified approach, samples were centrifuged at 500 × *g *for 5 min at 4°C to pellet intact cells and nuclei; importantly, no brake was applied during the deceleration of the centrifuge. The supernatant was decanted carefully and centrifuged at 16,000 × *g *in a Beckman microcentrifuge for 10 min at 4°C to pellet membranes. The pellet remaining from the low-speed spin, comprising predominantly nuclei, was lysed in 50 mM Tris-HCl pH 8.0, 150 mM NaCl, 1% Igepal CA-630, 0.5% sodium deoxycholate and 0.1% SDS plus protease and phosphatase inhibitors. In each case (conventional or modified), pellets from the high-speed centrifugation steps were then subjected to successive detergent extraction in Triton X-100 and octylglucoside to yield Triton-soluble (TS) and Triton-insoluble/octylglucoside-soluble (TI) fractions, as described in Methods.

Following determination of the protein concentration of fractions by MicroBCA assay, equal amounts of protein from each fraction were resolved by SDS-PAGE and transferred to nitrocellulose membranes. To verify the presence of protein in each fraction, membranes were stained with 0.1% (w/v) Ponceau S in 5% acetic acid (v/v) prior to blotting with the indicated antibodies. The staining pattern revealed both quantitative and qualitative differences in the overall protein profiles of TS and TI fractions generated by the two methods (Figure 1B). We confirmed the distribution of MyrAkt1 in TS, TI and nuclear (N) fractions by HA immunoblot, and observed robust enrichment of MyrAkt1 in TI fractions in both cases (Figure 1C). A small amount of MyrAkt1 was detected in the nuclear pellet from the low speed centrifugation, consistent with previous reports [32]. To determine the impact of the modified extraction procedure on protein distribution, we probed the membranes for selected proteins with defined subcellular localizations, including proliferating cell nuclear antigen (PCNA), a nuclear protein [33] and the small G-protein, G_iα2_, that resides in lipid rafts [34,35]. As shown in Figure 1C, PCNA, although present in the TI fraction following the conventional extraction method, was undetectable in TI fractions prepared with the modified approach. As anticipated, the nuclear fraction showed robust signal corresponding to PCNA. In contrast, G_iα2 _was present in TI fractions generated with either extraction method, consistent with its localization to DRMs. These data were reproducible across three independent biological replicates and similar findings were obtained with fractions isolated from LNCaP cells stably expressing the irrelevant gene (*LacZ*). We also probed for two additional proteins that were identified as putative raft-resident binding partners of MyrAkt1 transiently expressed in HEK293 cells (N.K.M. and R.M.A, unpublished observations): scaffold attachment factor B (SAFB), a protein implicated in transcriptional regulation and chromatin organization [36]; and heterogeneous ribonucleoprotein K (hnRNP K), a pleiotropic nucleic acid binding protein that regulates gene and protein expression [37,38]. Although SAFB was present in TI fractions generated by conventional means, it was absent from this material following elimination of nuclei, strongly suggesting that its presence in the TI fraction arose from nuclear contamination. In contrast, hnRNP K was detected in TS and TI fractions with both extraction methods, as well as in nuclei. These observations are consistent with localization of hnRNP K to multiple distinct subcellular sites [37].

To extend our analysis beyond characterization of specific proteins and to determine the potential utility of our modified fractionation procedure for proteomic applications, we used LC/MS as a read-out for the proteome compositions of TI fractions generated using either the conventional or modified methods. The TI fractions from the two extraction methods were subjected to in-solution tryptic digestion and mass spectrometry analysis, as described in Methods. According to the criteria outlined in Methods, 388 and 371 proteins were identified from the TI fractions isolated using the conventional and modified methods, respectively [see Additional file 1]. This proteomic analysis was not meant to be exhaustive. Instead we used the most confident protein identifications to a) ensure that the lipid raft isolation was successful and b) estimate the fraction of nuclear proteins. Common proteins identified in the TI fractions from both extraction methods included a number of proteins previously reported to be associated with DRMs, lipid rafts and/or caveolae (Table 1), such as flotillins [16,25,29,39,40], heterotrimeric G-protein subunits [25], components of the vacuolar ATP synthase protein family [16,25,29,41] and others [29,42-44]. Thus, the modified fractionation procedure did not alter the predicted composition of DRM fractions significantly and yielded fractions consistent with those isolated by density gradient-based methods.

**Table 1:** Proteins identified by tandem mass spectrometry.

**(A) Proteins associated with DRMs**			
**DRM proteins**			**References**
**Accession #**	**Score**	**Description**	
IPI00006211	175	VAMP (Vesicle-associated membrane protein)-associated protein B and C^a^	[42]
IPI00008453	122	Coronin-1C	[26]
IPI00013981	95	YES tyrosine kinase	[25]
IPI00015148	131	Ras-related protein Rap-1b	[40]
IPI00018511	95	Tubulin beta-4q chain	[40]
IPI00022418	285	Splice Isoform 1 of Fibronectin precursor	[26]
IPI00027438	388	Flotillin-1	[16, 25, 39]
IPI00029625	133	Flotillin-2	[16, 29, 39, 40]
IPI00030910	185	GPI-anchored protein p137^a^	[43]
IPI00030919	95	Mitogen-activated protein kinase kinase 1 interacting protein 1	[25], [40]
IPI00030939	185	Alpha subunit of GsGTP binding protein	[25]
IPI00216308	2152	Voltage-dependent anion-selective channel protein 1	[44]
IPI00220416	101	Ubiquinol-cytochrome c reductase complex 14 kDa protein	[29, 44]
IPI00221232	106	G-protein gamma-12 subunit variant	[25]
IPI00334190	552	Stomatin-like protein 2	[44]
IPI00337415	81	Guanine nucleotide-binding protein G(i) alpha-1 subunit	[25]
IPI00339269	163	Heat shock 70 kDa protein 6	[40]
IPI00440493	440	ATP synthase alpha chain, mitochondrial precursor	[40]
IPI00554701	72	Ubiquinol-cytochrome c reductase complex 7.2 kDa protein	[29, 44]
IPI00554711	603	Junction plakoglobin	[25]
IPI00641181	143	MARCKS-like 1	[28]

**(B) Nuclear proteins shown to exist in other subcellular locations**			
**Nuclear proteins**			
**Accession #**	**Score**	**Description**	**Evidence for shuttling**

IPI00000690	87	Splice Isoform 1 of Programmed cell death protein 8, mitochondrial precursor	Yes: [46]
IPI00012048	76	Nucleoside diphosphate kinase A	Yes: [47]
IPI00012442	190	Ras-GTPase-activating protein binding protein 1	Yes: [48]
IPI00016249	91	Fragile X mental retardation syndrome-related protein 1	Yes: [49]
IPI00016250	71	Fragile X mental retardation syndrome-related protein 2	Yes: [49]
IPI00021840	230	40S ribosomal protein S6	Yes: [50]
**IPI00023591**	**158**	**Transcriptional activator protein PUR-alpha**	**No:**
**IPI00024157**	**114**	**FK506-binding protein 3**	**No: **
IPI00027096	74	39S ribosomal protein L19, mitochondrial precursor	Yes: UniProt 49406
**IPI00027415**	**171**	**MLEL1 protein**	**No: **
IPI00028376	92	Mitochondrial import inner membrane translocase subunit TIM8 A	Yes: [51]
**IPI00032003**	**60**	**Emerin**	**No:**
IPI00042578	83	Similar to heterogeneous nuclear ribonucleoprotein A1 isoform a	Yes: [58]
IPI00165506	125	Polymerase delta interacting protein 2	Yes: [52]
IPI00215637	131	DEAD-box protein 3, X-chromosomal	Yes: [53]
IPI00215780	167	40S ribosomal protein S19	Yes: [54]
**IPI00217468**	**87**	**Histone H1.5**	**No:**
IPI00219330	206	Splice Isoform 5 of Interleukin enhancer-binding factor 3	Yes: [55]
IPI00328715	216	LYRIC protein	Yes: [56]
IPI00410693	94	Splice Isoform 1 of Plasminogen activator inhibitor 1 RNA-binding protein	Yes: [57]
IPI00456731	123	Similar to Laminin receptor 1	Yes: UniProt P08865

(A) The table illustrates proteins identified in DRM fractions generated using the modified fractionation method that have previously been described as present in or associated with DRMs isolated by other methods. (B) The table lists proteins identified in the modified DRM (TI) fraction that are indicated to be nuclear-localized based on Gene Ontology Cellular Component terminology, but that have been reported to exist in other locations. Proteins that are restricted to nuclei are shaded in grey.

^a^Likely DRM-associated.

**Figure 2:**
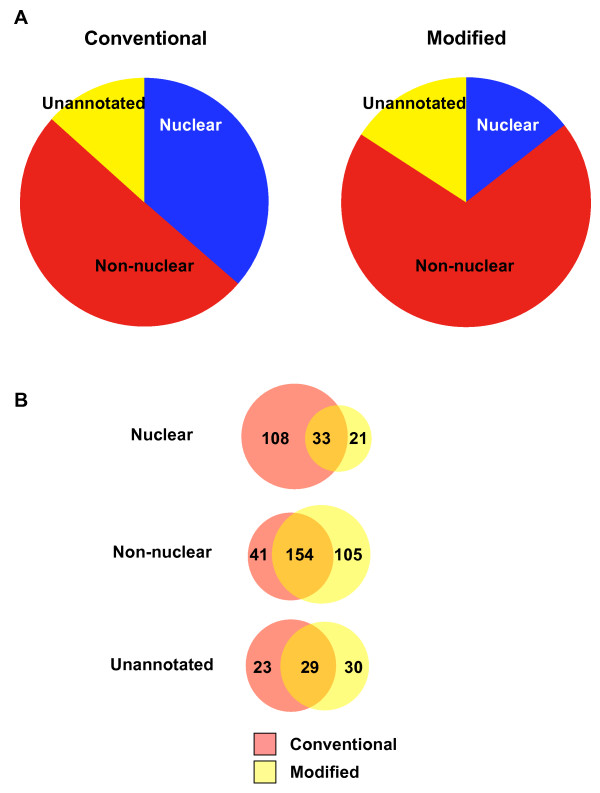
**Quantitative analysis of proteins in detergent-resistant membrane fractions analyzed by mass spectrometry**. **(A) **Proteins in DRM (TI) fractions generated using the conventional or modified methods were analyzed by tandem mass spectrometry and classified as either nuclear, non-nuclear or unannotated based on Gene Ontology cellular component terms. The pie-charts demonstrate the marked reduction in proteins identified as 'nuclear' with the modified extraction procedure. **(B) **Venn diagrams illustrating the distinct and overlapping protein compositions of TI fractions isolated by each method.

To assess the impact of the modified extraction procedure on the protein profiles obtained from each fraction, we classified proteins according to subcellular localization. In particular, we focused on whether proteins were nuclear or non-nuclear in nature. Datasets were annotated using Gene Ontology Cellular Component (GO-CC) terms with the cross-reference converter [45]. As shown in Figure 2A, we observed a striking difference in the number of nuclear proteins differentially identified with the two approaches. Specifically, the modified extraction procedure led to identification of many fewer nuclear proteins, with only 54 out of 371 proteins (15%) classified as nuclear. This was in marked contrast to the conventional method in which 141 out of 388 proteins (36%) were categorized as nuclear. At the same time, the number of proteins annotated as non-nuclear increased from 195 (50%) to 258 (70%). The Venn diagrams in Figure 2B clearly show that the decrease of nuclear proteins in the modified protocol is concomitant to the increase in non-nuclear proteins. These findings are in agreement with our findings by immunoblot analysis that the nuclear proteins PCNA and SAFB are no longer detectable in TI fractions generated using the modified fractionation technique.

Interestingly, of the 21 proteins annotated as nuclear in localization, that were found exclusively in the modified TI fraction, evidence from the literature and protein databases suggests that 16 of these are also present in additional subcellular locations, and may shuttle between the nucleus and other sites in the cell [46-58]. Thus, only 5 proteins (shaded in Table 1B), out of a total of 156 specifically identified in the modified TI fraction in this experiment, were present exclusively in nuclei. In addition, at least 60% of the 33 nuclear proteins identified with both approaches are also present in other subcellular locations. In contrast, only about 30% of the 108 nuclear proteins specifically identified in the conventional TI fraction are annotated to be present in additional locations.

These findings strongly suggest that the modified DRM extraction method generates fractions that are largely free of nuclear contamination. They also imply that, given the high frequency with which so-called 'nuclear proteins' are found in other organelles, due to shuttling between compartments, nuclear proteins are likely to be detected by most extraction procedures, even when performed under the most stringent conditions. Canonical nuclear proteins, such as nuclear hormone receptors, can shuttle between the cytoplasm and nuclear compartments as part of their hormone-dependent functions. Recently, the androgen receptor was found to process androgenic signals non-genomically by forming a complex with Akt1 in lipid raft microdomains [59]. The identification of such proteins in DRMs may thus reflect a legitimate physiologic process and not an experimental artifact.

## Conclusion

In summary, we describe a rapid, robust method for isolation of detergent-resistant membrane fractions that are compatible with downstream proteomic analyses with minimal additional processing. The present approach allows for a significant reduction of nuclear protein contamination of DRM samples. We believe the results presented here indicate that the DRM isolation procedure we describe can be applied with confidence to settings where a robust characterization of DRM constituents by mass spectrometry is desired, and also to more traditional biochemical methods involving antibody-based analyses.

## Methods

### Membrane preparations and isolation of detergent resistant membrane fractions

Populations of LNCaP/MyrHA-Akt1 or LNCaP/LacZ cells in log-phase growth were rinsed twice with phosphate-buffered saline (PBS), resuspended in Buffer M [50 mM HEPES pH 7.4, 10 mM NaCl, 5 mM MgCl_2_, 0.1 mM EDTA plus protease inhibitor cocktail (Complete Mini, Roche Applied Science), 1 mM Na_3_VO_4_, 1 mM NaF and 1 mM Na_4_P_2_O_7_.10 dH_2_O] and homogenized at 4°C using a Potter-Elvehjem tissue grinder (12 strokes, 1800 rpm). 10^7^-10^8 ^cells were used for each extraction method. High-speed (16,000 × g) pellets from either the conventional or modified approach were subjected to successive detergent extraction essentially as described [19,21,24]. Briefly, pellets were resuspended in Buffer A [25 mM 2-(*N*-morpholino)-ethanesulfonic acid, 150 mM NaCl, pH 6.5] and samples combined with an equal volume of Buffer A containing 2% Triton X-100, and phosphatase and protease inhibitors. Samples were incubated on ice for 60 min, centrifuged at 16,000 × g for 20 min at 4°C and supernatants collected as Triton-soluble (TS) material. Pellets were rinsed briefly with Buffer A and resuspended in Buffer B [10 mM Tris-Cl, pH 7.6, 150 mM NaCl, 60 mM β-octylglucoside and phosphatase and protease inhibitors]. Samples were incubated on ice for 30 min, centrifuged at 16,000 × g for 20 min at 4°C and supernatants collected as Triton-insoluble (TI) material.

### Tryptic Digestion and Mass Spectrometry Analysis

Four micrograms of each TI fraction was subjected to in-solution tryptic digestion. Three MS experiments were performed: TI fraction from the conventional extraction procedure; TI fraction from the modified extraction procedure; and extraction buffer control with a single replicate for each. Approximately 2 μL protein extracts or extraction buffer as control, were mixed with 8 μL 0.2% RapiGest™ SF (Waters Corporation, Milford, MA) in 50 mM ammonium bicarbonate, boiled for 5 min and cooled to room temperature. Dithiothreitol was added to 5 mM final concentration, samples heated at 60°C for 30 min and cooled to room temperature. Iodoacetamide was added to 15 mM final concentration and samples were incubated protected from light for 30 min prior to addition of 100 ng MS-grade trypsin and further incubation at 37°C for 2 h. To hydrolyze the RapiGest™ surfactant, trifluoroacetic acid was added to a final concentration of 0.5% and samples were incubated at 37°C for 45 min. Following centrifugation at 14,000 × *g *for 10 min, the supernatants were transferred to screw-top vials. Acetonitrile and acetic acid were added to a final concentration of 7.5% and 1.5%, respectively. Samples were analyzed by online C_18 _nanoflow reversed-phase HPLC (Eksigent nanoLC·2D™) hyphenated to a Thermo Scientific LTQ Orbitrap mass spectrometer. The digest was loaded onto a 100 μm i.d. × 15 cm C_18 _column and the peptides were separated at 200 nL/min with 80 min gradients from 5 to 31.5% acetonitrile in 0.4% formic acid. Survey spectra were acquired in the orbitrap with the resolution set to a value of 30,000. Up to five of the most intense ions per cycle were fragmented and analyzed in the linear trap.

Database searching was performed using the Mascot search engine (Matrix Science, v.2.1). All MS datasets were searched against the International Protein Index (IPI) human protein database of the International Protein Index (v3.10; 57479 sequences). Protein modifications were selected as carbamidomethyl (C) (fixed) and oxidation (M) (variable). Up to one missed cleavage was allowed. The mass tolerance was set as ± 20 ppm for MS spectra and ± 0.5 Da for MS/MS spectra. The following criteria were used to generate a high-confidence data set: at least two peptides must be identified for each protein, with a score not less than 33, corresponding to a significance level of p < 0.01 as determined by reverse database searching. Known contaminants (e.g. keratins and trypsin) were removed from the protein lists. Applying these parameters, no false positives were identified through searching the reversed IPI_Human database.

## Authors' contributions

RMA conceived of the study, performed the fractionation and immunoblot analyses and drafted the manuscript. WY performed mass spectrometry and data analysis on DRM fractions. DDV performed immunofluorescence staining. NKM provided data on localization of hnRNP K and SAFB to detergent-resistant membrane and nuclear fractions. HS assisted with mass spectrometry analysis and data mining and helped to draft the manuscript. All authors have read and approved the final manuscript.

## Supplementary Material

Additional file 1 MS Data Summary. The table summarizes MS data obtained from DRM fractions isolated using either the conventional or modified extraction procedures. Click here for file
